# Long non-coding RNA VCAN-AS1 promotes the malignant behaviors of breast cancer by regulating the miR-106a-5p-mediated STAT3/HIF-1α pathway

**DOI:** 10.1080/21655979.2021.1960774

**Published:** 2021-08-09

**Authors:** Peng Du, Kaifeng Luo, Guoyong Li, Jisheng Zhu, Qi Xiao, Yong Li, Xingjian Zhang

**Affiliations:** Department of General Surgery, The First Affiliated Hospital of Nanchang University, Nanchang, Jiangxi, China

**Keywords:** Breast cancer, VCAN-AS1, miR-106a-5p, STAT3, HIF-1α, ceRNA

## Abstract

An accumulating number of studies have found that long noncoding RNAs (lncRNAs) participate in breast cancer (BC) development. LncRNA VCAN-AS1, a novel lncRNA, has been confirmed to regulate the progression of gastric cancer, while its role in BC is elusive. Here, our results illustrate that VCAN-AS1 is overexpressed in BC tissues and cells, while miR-106a-5p was downregulated and negatively correlated with VCAN-AS1. In addition, high VCAN-AS1 expression and low miR-106a-5p expression were closely correlated with poor overall survival in BC patients. Functional experiments confirmed that VCAN-AS1 overexpression notably accelerated BC cell proliferation, migration, invasion, and epithelial–mesenchymal transition (EMT) and enhanced tumor cell growth while also suppressing cell apoptosis. However, overexpression of miR-106a-5p had the opposite effects. In addition, rescue experiments confirmed that overexpression of VCAN-AS1 inhibited the tumor-suppressive effects mediated by miR-106a-5p. Mechanistically, through bioinformatics analysis, we found that VCAN-AS1 functions as a competitive endogenous RNA (ceRNA) of miR-106a-5p, which targets the 3ʹ untranslated region (UTR) of signal transducer and activator of transcription 3 (STAT3). Further experiments indicated that miR-106a-5p downregulated the STAT3/hypoxia-inducible factor-1alpha (HIF-1α) pathway, while activating the STAT3 pathway reversed miR-106a-5p-mediated antitumor effects. Collectively, our data suggest that VCAN-AS1 is upregulated in breast cancer and promotes its progression by regulating the miR-106a-5p-mediated STAT3/HIF-1α pathway. This study provides a new target for BC therapy.

## 1. Introduction

Breast cancer (BC) is the most frequently diagnosed cancer in the world among women [1]. Despite recent advances in early diagnosis and effective treatment, BC in some patients progresses to the metastatic stage after therapy for unknown reasons [2,3]. Therefore, it is essential to search for novel molecules to understand the progression of breast cancer.

LncRNAs are noncoding RNAs that are over 200 bases in length [4]. Despite the lack of protein-coding capacity, lncRNAs participate in gene expression regulation and are generally considered essential regulators of cancers [5,6]. Various lncRNAs play different regulatory roles in BC. lncRNA SNHG1 inhibits miR-573, resulting in increased LIM domain only 4 (LMO4) content, which accelerates BC cell proliferation and migration [7]. Additionally, lncRNA HOTTIP directly binds to miR-148a-3p and inhibits WNT1, leading to inactivation of the Wnt/β-catenin signaling pathway and aggravated BC development [8]. VCAN antisense RNA 1 (VCAN-AS1) (GeneID: 105,379,054) is a lncRNA transcribed from a gene located on 5q14.3. Recent studies have revealed that VCAN-AS1 participates in the regulation of gastric cancer development [9,10]. However, the role of VCAN-AS1 in BC development remains unclear.

MicroRNAs (miRNAs), which are another type of noncoding RNA, greatly influence a variety of biological processes, such as cell differentiation, intracellular homeostasis, genomic imprinting and organogenesis [11]. It can be transcribed and processed similar to mRNA, but it does not code for proteins, and it affects various biological processes by regulating mRNA expression [12]. MiRNAs are abnormally expressed in various human diseases, including BC, and play an important role [13]. MiR-106a-5p, a miRNA, has been proven to be abnormally expressed in cancer patients and regulates cancer progression. For instance, miR-106a-5p was downregulated in clear cell renal cell carcinoma (RCC) and inhibited tumor cell growth and colony formation ability [14]. In addition, miR-106a-5p sensitizes osteosarcoma cells to cisplatin treatment by targeting signal transducer and activator of transcription 3 (STAT3) [15].

STAT3 is a well-characterized oncogene that affects various biological processes, including promoting cell proliferation and survival by regulating Cyclin D1 and Bcl-2, inducing tumor invasion and metastasis by regulating E-cadherin and MMP-9, and promoting angiogenesis by HIF-1α expression [16]. Moreover, STAT3 is a well-identified oncogenic regulator in breast cancer [17]. Hypoxia-inducible factor-1alpha (HIF-1α) principally mediates the transcriptional response of mammalian cells to hypoxia, which normally degenerates under normoxic circumstances and is stabilized in the hypoxic microenvironment [18]. A wealth of evidence proves that high expression levels of HIF-1α in solid tumors and during tumor growth are significantly limited after knocking out HIF-1α, which indicates that HIF-1α plays a vital role in cancer development [19,20]. Importantly, several studies pointed to the interaction of HIF-1α with other signaling proteins, such as STAT3, for transactivation of HIF-1α target genes [16,21–23].

In recent years, studies have found that lncRNAs function as competitive endogenous RNAs (ceRNAs) by sponging miRNAs, which then target the 3ʹ-UTR of mRNAs. The lncRNA-miRNA-mRNA network prominently modulates tumor development. Thus, we hypothesized that there is a regulatory axis of VCAN-AS1-miR-106a-5p-STAT3/HIF-1α in BC development. In the present study, we performed *in vitro* and *in vivo* experiments to identify the role of VCAN-AS1 in BC development, and we explored related regulatory mechanisms with the purpose of providing a novel target for BC treatment strategies

## 2. Materials and methods

### 2.1. Clinical specimen collection

Tumor tissues and healthy paracancerous tissues were collected from 40 pairs of BC patients undergoing surgical treatment at The First Affiliated Hospital of Nanchang University from January 2019 to April 2019. All patients were diagnosed with BC by pathologists at The First Affiliated Hospital of Nanchang University after pathological examination, and no tumor cells were found in the adjacent normal tissues. Once collected, each sample was divided: one part was fixed in paraformaldehyde, and the other part was immediately frozen and stored at −80°C for further use. General clinical data of the patients were collected, and patient prognosis was assessed after a follow-up period of 5–48 months. All patients who had not received radiotherapy, chemotherapy or other treatments prior to diagnosis agreed to be involved in this study and gave signed informed consent. Detailed clinicopathological characteristics of the patients are listed in Table 1. This study was approved by the ethics committee of The First Affiliated Hospital of Nanchang University (Approval number: NCU-2019-023).

**Table 1.** The clinicopathological characteristics of the 40 breast cancer patients included

	Cases (%)
Age	
≤ 50	21 (52.5%)
> 50	19 (47.5%)
Tumor size	
≤ 2cm	17 (42.5%)
> 2cm	23 (57.5%)
ER status	
Negative	14 (35.0%)
Positive	26 (65.0%)
PR status	
Negative	12 (30.0%)
Positive	28 (70.0%)
HER-2 status	
Negative	29 (72.5%)
Positive	11 (27.5%)
Lymphatic metastasis	
Negative	22 (55.0%)
Positive	18 (45.0%)
TNM stage	
I/II	24 (60.0%)
III/IV	16 (40.0%)
Distant metastasis	
Negative	31 (77.5%)
Positive	9 (22.5%)

Abbreviations: TNM: tumor–node–metastasis; ER: estrogen receptor;PR: progesterone receptor; HER-2: receptor tyrosine-protein kinase erbB-2.

### 2.2. Cell culture

All BC cell lines (MCF7, MDA-MB-453, MDA-MB-231 and BT-549) and the human normal mammary epithelial cell line (MCF-10A) were purchased from the China Center for Type Culture Collection (Wuhan, China). Cells were grown in Dulbecco’s minimal essential medium/Ham’s F12 (DMEM/F12) medium (Thermo Fisher Scientific, Shanghai, China), which contained 10% fetal bovine serum and were incubated with 5% CO_2_ at 37°C. The medium was changed once every other day. When the cell confluence reached 80%, the cells were trypsinized with 0.25% trypsin.

### 2.3. Cell transfection

RiBoBio (Shanghai, China) provided the pcDNA-*VCAN-AS1* overexpression vector (*VCAN-AS1*) and its negative control (vector), small inferencing RNA targeting VCAN-AS1 (si-VCAN-AS1#1, si-VCAN-AS1#2) and its negative control (si-NC), and miR-106a-5p mimics and its negative control (miR-NC). BC cell lines (MCF7, MDA-MB-453, MDA-MB-231 and BT-549) were digested using 0.25% trypsin, centrifuged (170 g for 5 min at room temperature) and collected. Next, BC cell lines (MCF7, MDA-MB-453, MDA-MB-231 and BT-549) were **seeded** in 24-well plates (1 × 10^5^ cells each). After 24–36 h, the cell growth was stabilized, and the cells reach 80% confluency. VCAN-AS1, vector, si-VCAN-AS1, si-NC, miR-106a-5p mimics or miR-NC were transfected into MCF7 and MDA-MB-453 cells using FuGENE®HD Transfection Reagent (Roche, Shanghai, China) in accordance with the manufacturer’s instructions. Then, the cells were cultured at 37°C (5% CO_2_) for 24 h. Then, the culture medium was replaced with fresh culture medium. After an additional incubation at 37°C (5% CO_2_) for 24 h, total cell RNA was extracted for real-time fluorescence quantitative PCR (RT-PCR) to assess the altered VCAN-AS1 and miR-106a-5p expression in the transfected cells.

### 2.4. RT-PCR

BC tissues and adjacent normal tissues were collected and lysed in TRIzol reagent (Invitrogen, Carlsbad, CA, USA). Total RNA was extracted using the chloroform-isopropanol-ethanol method, and 75% ethanol was used for RNA precipitation. miRNA was extracted using a PureLink® miRNA Isolation Kit (Thermo Fisher Scientific, Shanghai, China). The concentration and purity of the total RNA were determined on an ultraviolet spectrophotometer (UV-1600PC, Mapada). The RevertAid First Strand cDNA Synthesis Kit (Thermo Fisher Scientific, Waltham, MA, USA) was then used to perform reverse transcription of 2 μg of total RNA from each group. The reaction conditions were as follows: 70°C for 10 min; 5 min on ice; 42°C for 60 min; 95°C for 5 min; and 0°C for 5 min. The reverse transcription reaction of 2 μg of microRNA was performed using the TaqMan™ MicroRNA Reverse Transcription Kit (Thermo Fisher Scientific, Shanghai, China). Then, the obtained cDNA was amplified by quantitative fluorescence PCR on an Applied Biosystems™ 7500 Real-time fluorescent quantitative PCR system (Thermo Fisher Scientific). The 25 μL reaction contained 500 ng of each cDNA template, 0.25 pmol/μL of reverse and forward primers, and 12.5 μL of 2× SYBR Green qPCR Master Mix (MedChemExpress, NJ, USA). The primers were designed and synthetized by Sangon Biotech (Shanghai, China). The 2^−ΔΔCt^ method was adopted to determine the relative expression of VCAN-AS1 and miR-106a-5p. U6 was used as the endogenous control for miR-106a-5p, and GAPDH was used as the endogenous control for the other genes. The primer sequences of each molecule are shown in Table 2.

**Table 2.** The primer sequences for RT-PCR

Gene name	Forward primer	Reverse primer
VCAN-AS1	5ʹ-TGTTTTCCTTGGCTTTTGGA-3’	5ʹ-GCTTTTCTCCACCCCACTTT-3’
miR-106a-5p	5ʹ-AACAATCAAAGTGCTGTTCGTGC-3’	5ʹ-CAGTGCAGGGTCCGAGGT-3’
STAT3	5ʹ-CCAGTCAGTGACCAGGCAGAAG-3’	5ʹ-GCACGTACTCCATCGCTGACA-3’
U6	5ʹ-ATTGGAACGATACAGAGAAGATT −3’	5ʹ-GGAACGCTTCACGAATTTG-3’
GAPDH	5ʹ-GTGCTTTGACAAATCCCATCTGA-3’	5ʹ-GTTACTGTCCCGGATCTTGTCCA-3’

### 2.5. Western blot

Total protein was extracted from the mouse tissues or cells using radioimmunoprecipitation assay (RIPA) lysis buffer (Beyotime, Shanghai, China). Then, a bicinchoninic acid (BCA) protein assay kit (Beyotime, Shanghai, China) was used to determine protein concentration. For each group, 20 μg of total protein was separated by sodium dodecyl sulfate-polyacrylamide gel electrophoresis (SDS-PAGE) and transferred to PVDF membranes (Millipore, USA) at a constant electric current of 300 mA. This was followed by blocking with a TBST solution containing 5% skim milk (2 h, room temperature), and the PVDF membranes were incubated with a primary antibody overnight at 4°C. The primary antibodies included Caspase3 (Abcam, ab13847, 1:1000), Bax (Abcam, ab32503, 1:1000), Bcl-2 (Abcam, ab32124, 1:1000), HIF-1α (Abcam, ab51608, 1:1000), STAT3 (Abcam, ab119352, 1:1000), E-cadherin (Proteintech, Cat. No. 20,874-1-AP, 1:1000), Vimentin (Proteintech, Cat. No. 10,366-1-AP, 1:1000), N-cadherin (Proteintech, Cat. 22,018-1-AP, 1:1000), β-actin (Proteintech, Cat. No. 60,008-1-Ig, 1:1000). Afterward, we rinsed the membranes with TBST 4 times (8 min each) and incubated them for 1.5 h with either HRP-conjugated Affinipure goat anti-mouse IgG (H + L) secondary antibody (Proteintech, SA00001-1, 1:5000) or HRP-conjugated Affinipure goat anti-rabbit IgG (H + L) secondary antibody (Proteintech, SA00001-2, 1:5000). Finally, we washed the membranes with TBST 4 times (8 min each time). X-ray development was performed using a Thermo Pierce ECL Western Blot Substrate kit.

### 2.6. Transwell assay

Cell migration and invasion were examined using 24-well chambers with 8 µm pore size membranes (Corning, Beijing, China) without (migration) or with Matrigel (invasion) as previously described [6]. Briefly, MCF7 and MDA-MB-453 cells underwent 0.25% trypsinization, centrifugation, and resuspension and were dispersed in single wells of a 24-well culture plate. A total of 5 × 10^4^ cells were suspended in the top chamber with 200 μL of FBS-free medium, and 500 μL of medium with 10% FBS was placed in the bottom chamber. For the invasion analysis, Matrigel (50 µl; BD Biosciences) was used to precoat the membrane surface. After incubation at 37°C for 24 h, the invading cells were fixed with methanol and then stained with crystal violet (0.5%) for 20 min at room temperature. After rinsing under running water, cells were counted under an inverted microscope. We conducted all experiments in triplicate and repeated each experiment three times.

### 2.7. Detection of cell proliferation and viability

MCF7 and MDA-MB-453 cells were seeded into 96-well plates and cultured for 24 h (1 × 10^3^ cells/well). Following the manufacturer’s instructions, BrdU Cell Proliferation ELISA Kit (colorimetric) (Abcam, ab126556) and Cell Counting Kit-8 (CCK8, MedChemExpress, Cat. No. HY-K0301) were used to examine cell proliferation and viability. The OD450 value was detected using a spectrophotometer (BIO-RAD, CA, USA) after the cells were treated with reagents.

### 2.8. Colony formation assay

A colony formation assay was performed to evaluate BC cell proliferation according to a previous study [23]. MCF7 and MDA-MB-453 cells were seeded into 60 mm dishes (with 300 cells per dish). Then, the cells were cultured in an incubator with 5% CO_2_/95% air (at 37°C) for 14 days. Then, methanol was used to fix the colonies for 15 min. After being washed with PBS 3 times, 0.1% crystal violet was used to stain the colonies for 15 min at room temperature. Finally, the colonies were observed and counted under a light microscope.

### 2.9. Tumor xenograft model

A tumor xenograft model was used to evaluate the role of VCAN-AS1 in BC cell growth as previously described [24]. Ten female BALB/c nude mice (4–6 weeks old, female, the Laboratory Animal Center of Wuhan University) were randomly divided into two groups (n = 5 per group), vector and VCAN-AS1. MCF7 cells transfected with VCAN-AS1 or vector were trypsinized to generate a single-cell suspension. The cell count was adjusted to 5× l0^6^/mL. MCF7 cells were treated with Matrigel (5 mg/ml) to enhance the rate of tumor formation. The left and right sides of each nude mouse were injected with 0.1 mL of 5× 10^6^/mL of the single-cell suspension, and the diet, activity and general condition of the nude mice were observed. Beginning on the 14th day, the tumor volume was measured according to the equation: volume = long diameter × short diameter^2^/2. The tumors were measured continuously for 5 weeks. Then, the mice were sacrificed, and the tumor weights were recorded. All animal experiments were approved by the Animal Care and Use Committee of the First Affiliated Hospital of Nanchang University and were in accordance with the National Guide for the Care and Use of Laboratory Animals.

### 2.10. Dual luciferase activity assay

A dual luciferase activity assay was performed to investigate the binding relationship between miR-106a-5p and VCAN-AS1 or STAT3 [23]. Vectors containing luciferase reporter sequences (including VCAN-AS1-WT, VCAN-AS1-MUT, STAT3-WT and STAT3-MUT) were constructed by Promega (Madison, WI, USA), in which VCAN-AS1-wt and STAT3-WT contained binding sites for miR-106a-5p. The binding sites of VCAN-AS1, STAT3 and miR-106a-5p were predicted through starBase, and neither VCAN-AS1-MUT nor STAT3-MUT had miR-106a-5p binding sites. MCF7 and MDA-MB-453 cells (4.5 × 10^4^) were seeded in a 48-well plate and cultured until 70% confluence. Then, Lipofectamine® 2000 (Invitrogen) was used to cotransfect VCAN-AS1-WT, VCAN-AS1-MUT, STAT3-WT or STAT3-MUT into MCF7 and MDA-MB-453 cells with miR-106a-5p mimics or negative control constructs. After 48 h of transfection, we evaluated the luciferase activity using the Dual-Luciferase Reporter Assay System (Promega). We conducted all experiments in triplicate and repeated each experiment three times.

### 2.11. RNA immunoprecipitation (RIP) assay

An RIP assay was performed using a Magna RIP™ RNA Binding Protein Immunoprecipitation Kit (Millipore, Bedford, MA, USA) [6]. After the transfected MCF7 and MDA-MB-453 cells were harvested, the cells were lysed using RIP lysis buffer (Solarbio, Beijing, China). Next, we added magnetic beads (Invitrogen) to RIP lysis buffer (Solarbio) and then conjugated the beads with anti-Ago2 (Abcam) or anti-IgG (Abcam) (overnight, 4°C). After digestion with proteinase K (Absin, Shanghai, China) the immunoprecipitated RNA was acquired and then quantified by RT-PCR.

### 2.12. Flow cytometry

MCF7 and MDA-MB-453 cells were transfected with miR-106a-5p and/or VCAN-AS1 vectors and treated with 0.25% trypsin. Annexin V-FITC double staining (BD Biosciences, New Jersey, USA) was used to detect cell apoptosis [9]. Briefly, after being rinsed with cold PBS, the cells were resuspended in binding buffer (100 mmol/L NaCl, 25 mmol/L CaCl_2_, 100 mmol/L HEPES, pH 7.4) and stained with Annexin V-FITC/PI (15 min, room temperature). Finally, flow cytometry was carried out on a BD FACSCelesta™ Flow Cytometer (BD company) to measure the cell apoptosis rate.

### 2.13. Data processing

All data are presented as the mean ± standard deviation (x ± s). Statistical analysis of data was implemented utilizing SPSS 19.0 (SPSS Inc., Chicago, IL, USA). After testing the normality of the data, univariate analysis of variance was adopted for data comparison between groups followed by Tukey’s test; Pearson correlation test was employed for analyzing the relationship between miR-106a-5p and VCAN-AS1 in BC tissues; difference analysis between the two groups was carried out by Student’s t-test. Kaplan–Meier survival curves were applied to observe the influence of VCAN-AS1 and miR-106a-5p expression differences on the prognosis of BC patients. Differences were considered statistically significant at *P* < 0.05.

## 3. Results

### 3.1. VCAN-AS1 and miR-106a-5p expression characteristics in BC tissues and cells

**Figure 1.**
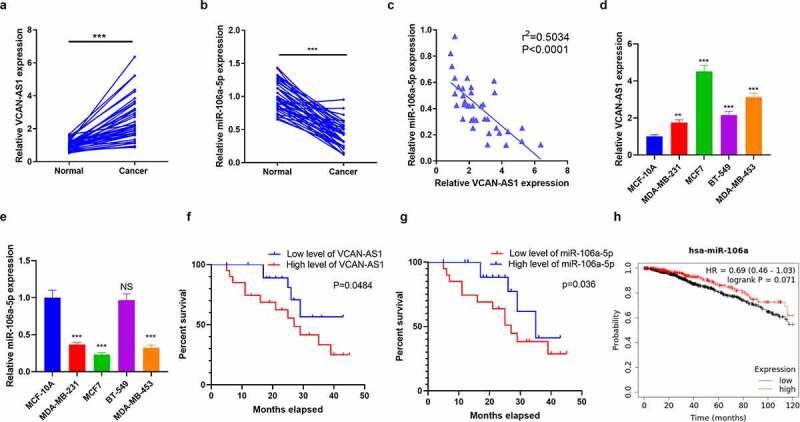
Expression characteristics of VCAN-AS1 and miR-106a-5p in BC tissues and cells

To investigate the effect of VCAN-AS1 and miR-106a-5p on the modulation of BC development, we collected 40 pairs of BC and adjacent normal tissues. The expression levels of VCAN-AS1 and miR-106a-5p were assessed by RT-PCR. The results illustrated that VCAN-AS1 levels in BC tissues were upregulated (compared with adjacent normal tissues, p < 0.001, Figure 1(a)), while miR-106a-5p was downregulated (compared with adjacent normal tissues, p < 0.001, Figure 1(b)). The results of linear regression analysis showed a significant negative correlation between VCAN-AS1 and miR-106a-5p in BC tissues (R^2^ = −0.5034, p < 0.0001, Figure 1(c)). Next, both VCAN-AS1 and miR-106a-5p levels in BC cell lines (MCF7, MDA-MB-453, MDA-MB-231 and BT-549) and a human normal mammary epithelial cell line (MCF-10A) were compared. The results showed that VCAN-AS1 levels were markedly enhanced in BC cell lines, with the highest level in MCF7 and MDA-MB-453 cells (Figure 1(d)), while miR-106a-5p was downregulated in BC cell lines (compared with MCF-10A) and had lower levels in MCF7 and MDA-MB-453 cells (Figure 1(e)). Furthermore, Kaplan–Meier plotter survival curves were used to analyze the relationship between VCAN-AS1 and miR-106a-5p and BC patient prognosis. The cutoff value of the VCAN-AS1 and miR-106a-5p level was the median. These results revealed that a high VCAN-AS1 level was related to worse survival in BC patients (p = 0.0484, Figure 1(f)), while a low miR-106a-5p level was associated with worse survival in BC patients (p = 0.036, Figure 1(g)). In addition, we discovered that lower miR-106a-5p expression was also associated with poorer survival of BC patients (p = 0.071, Figure 1(h)) [analyzed by Kaplan–Meier Plotter (http://kmplot.com/analysis/)]. Thus, both VCAN-AS1 and miR-106a-5p may facilitate the regulation of BC development and may interact with each other.

Forty pairs of BC tissues and healthy tissues adjacent to cancer were collected. (a-b). RT-PCR was carried out to detect VCAN-AS1 (a) and miR-106a-5p (b) expression levels. (c) Linear regression analysis of the correlation between VCAN-AS1 and miR-106a-5p in BC tissues. (d-e) RT-PCR was used to compared VCAN-AS1 and miR-106a-5p levels in MCF7, MDA-MB-453, MDA-MB-231 and BT-549 as well as human normal mammary epithelial cell line (MCF-10A). (f-g) The relationship between VCAN-AS1 (f), miR-106a-5p (g) and the prognosis of BC patients was analyzed by Kaplan–Meier Plotter survival curve. (h) Analysis of miR-106a-5p expression with the overall survival of BC patients by Kaplan–Meier Plotter (http://kmplot.com/analysis/). NS, **, *** indicates *p* > 0.05, *p* < 0.01 and *p* < 0.001 vs. the Normal or MCF-10A group, respectively.

### 3.2. VCAN-AS1 targeted miR-106a-5p

**Figure 2.**
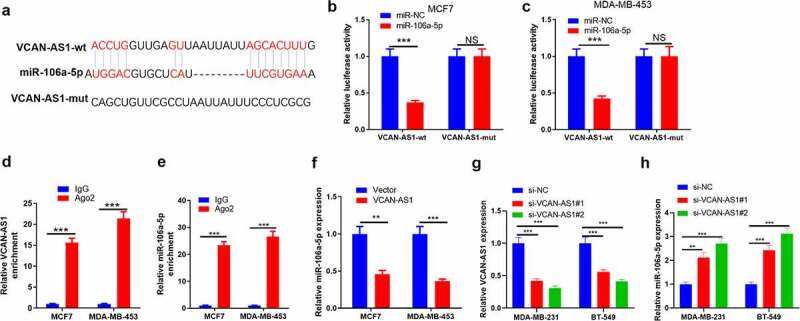
VCAN-AS1 targets miR-106a-5p

After searching the LncBase v.2 database, we found that miR-106a-5p contained complementary pairing sites with VCAN-AS1 (Figure 2(a)). To verify the interaction between the two, we carried out dual luciferase activity experiments and RIP experiments. The results showed that miR-106a-5p remarkably reduced the luciferase activity of transfected VCAN-AS1-WT cells (p < 0.001) but had no significant effect on the luciferase activity of transfected VCAN-AS1-MUT cells (p > 0.05, Figure 2(b,c)). In addition, compared with the anti-IgG group, more VCAN-AS1 and miR-106a-5p were enriched in the anti-Ago2 group (p < 0.001, Figure 2(d,e)). Furthermore, we transfected VCAN-AS1-overexpressing vectors into MCF7 and MDA-MB-453 cells and found that the miR-106a-5p level was significantly inhibited compared with that in the vector group (p < 0.01, Figure 2(f)). Furthermore, we detected miR-106a-5p levels in the downregulated cell models of VCAN-AS1 (p < 0.001, Figure 2(g)). We found that inhibiting VCAN-AS1 markedly promoted miR-106a-5p levels (p < 0.01, Figure 2(h)). Thus, VCAN-AS1 is an endogenous competitive RNA of miR-106a-5p and inhibits miR-106a-5p expression.

(a) Through LncBase v.2 database (http://starbase.sysu.edu.cn/), it was predicted that miR-106a-5p contains a site that is complementary to VCAN-AS1. (b-c) Using dual luciferase activity experiments to verify the regulatory relationship between miR-106a-5p and VCAN-AS1. (d-e) RT-PCR was carried out to detect the enrichment levels of VCAN-AS1 (d) and miR-106a-5p (e) in RIP lysates. (f) VCAN-AS1 overexpressing vectors were transfected into MCF7 and MDA-MB-453, and the level of miR-106a-5p in the cells was evaluated via RT-PCR. (g) si-VCAN-AS1 or si-NC vectors were transfected into MDA-MB-231 and BT-549. h. The level of miR-106a-5p in the cells was evaluated via RT-PCR. NS means *p* > 0.05, ** means *p* < 0.01, *** means *p* < 0.001.

### 3.3. Effects of VCAN-AS1 and miR-106a-5p on BC cell proliferation and apoptosis

**Figure 3.**
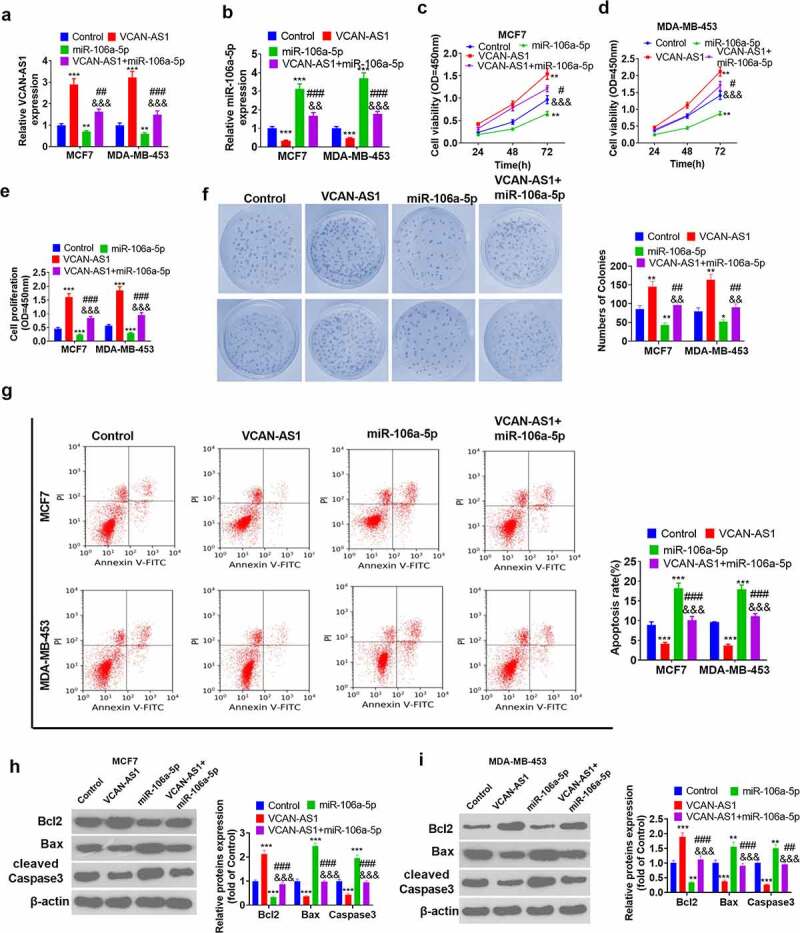
Effects of VCAN-AS1 and miR-106a-5p on BC cell proliferation and apoptosis

To explore the regulation of the malignant phenotype in BC cells by both VCAN-AS1 and miR-106a-5p, we transfected VCAN-AS1 overexpression plasmids and/or miR-106a-5p mimics into MCF7 and MDA-MB-453 BC cells. The results showed that the level of miR-106a-5p in the VCAN-AS1 + miR-106a-5p group was notably downregulated compared with that in the miR-106a-5p group (p < 0.01, Figure 3(a,b)). Then, CCK8 and BrdU experiments were performed to assess cell viability and proliferation. The results illustrated that cell viability, proliferation, and colony formation increased in the VCAN-AS1 group, while that of the miR-106a-5p group decreased (compared with the control group) (p < 0.05, Figure 3(c,f)). Interestingly, compared with that in the miR-106a-5p group, cell viability and proliferation in the VCAN-AS1 + miR-106a-5p group were increased (p < 0.05, Figure 3(c,f)). In addition, cell apoptosis was detected through flow cytometry and western blot analysis. The results showed that the upregulation of VCAN-AS1 limited the cell apoptosis rate, upregulated Bcl-2 expression and inhibited the expression of Caspase3 and Bax, while upregulation of miR-106a-5p enhanced cell apoptosis (p < 0.001, Figure 3(g,i)). Moreover, the apoptosis level of the cells in the VCAN-AS1 + miR-106a-5p group was remarkably inhibited compared with that of the miR-106a-5p group (p < 0.01, Figure 3(g,f)). Thus, VCAN-AS1 promotes the viability of BC cells and suppresses cell apoptosis. Meanwhile, upregulated VCAN-AS1 also exerts its effect by inhibiting miR-106a-5p.

BC cells MCF7 and MDA-MB-453 were transfected with VCAN-AS1 overexpression plasmid and/or miR-106a-5p mimic, respectively (a-b). RT-PCR was performed to measure the expression of VCAN-AS1 (a) and miR-106a-5p (b). (c-d) Cell viability was detected using the CCK8 method. (e) The cell proliferation ability was examined via the BrdU method. (f) The cell colony ability was examined via the colony formation method. (g) Flow cytometry detection of cell apoptosis rate. (h-i) The expression of apoptosis-related proteins Bcl-2, Caspase 3 and Bax were measured by western blot. ** *p* < 0.01, *** *p* < 0.001 vs. control group, # *p* < 0.05, ## *p* < 0.01, ### *p* < 0.001 vs. VCAN-AS1 group, && *p* < 0.01, &&& *p* < 0.001 vs. miR-106a-5p group.

### 3.4. Effects of VCAN-AS1 and miR-106a-5p on BC cell migration, invasion and EMT

**Figure 4.**
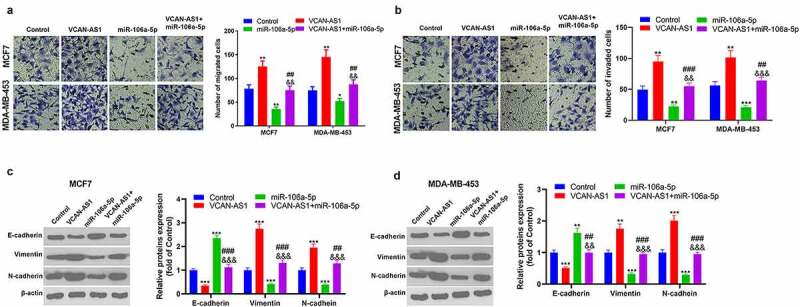
Effect of VCAN-AS1 and miR-106a-5p on BC cell migration, invasion and EMT (a-b)

Furthermore, the altered migration, invasion and EMT of MCF7 and MDA-MB-453 cells were detected using a transwell assay and western blot analysis. The results revealed that the migration and invasion ability of cells in the VCAN-AS1 group was enhanced, while that of cells in the miR-106a-5p group was weakened compared with the control group (p < 0.05, Figure 4(a,b)). At the same time, compared with that in the miR-106a-5p group, the cell migration and invasion ability in the VCAN-AS1 + miR-106a-5p group was increased (p < 0.01, Figure 4(a,b)). In addition, the expression of EMT markers (E-cadherin, vimentin and N-cadherin) was detected by western blot. The results showed that VCAN-AS1 overexpression downregulated E-cadherin in cells and upregulated Vimentin and N-cadherin, while miR-106a-5p overexpression had the opposite effects (p < 0.01, Figure 4(c,d)). Moreover, compared with the miR-106a-5p group, the EMT of cells in the VCAN-AS1 + miR-106a-5p group was significantly enhanced (p < 0.01, Figure 4(c,d)). Thus, VCAN-AS1 promotes BC cell migration, invasion and EMT, and this effect is largely achieved by inhibiting miR-106a-5p.

Transwell test was employed to detect MCF7 and MDA-MB-453 cell migration (a), invasion ability (b), (c-d). Changes in EMT-related proteins (E-cadherin, Vimentin and N-cadherin) were measured via western blot. * *p* < 0.05, ** *p* < 0.01, *** *p* < 0.001 vs. control group, ## *p* < 0.01, ### *p* < 0.001 vs. VCAN-AS1 group, && *p* < 0.01, &&& *p* < 0.001 vs. miR-106a-5p group.

### 3.5. STAT3 is the downstream target of miR-106a-5p

**Figure 5.**
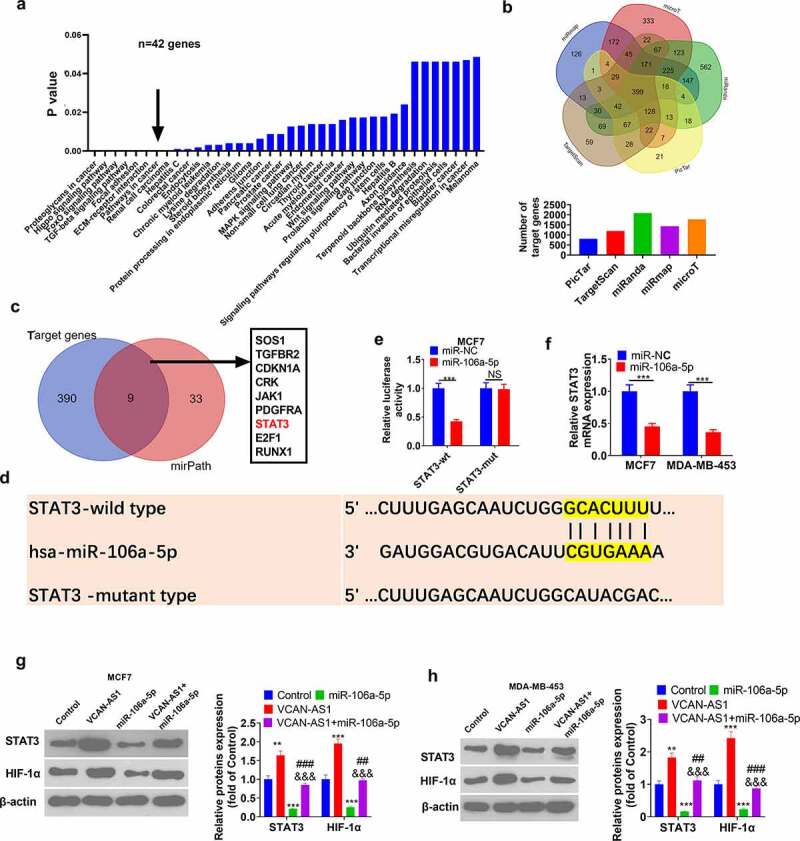
STAT3/ HIF-1α is the downstream target of miR-106a-5p

For more profound exploration of the downstream molecular mechanism of miR-106a-5p, we predicted the potential signaling pathways and molecular targets of miR-106a-5p through mirPath v.3 and starBase, respectively. The results revealed that miR-106a-5p potentially regulates 38 significant pathways (p < 0.05), and ‘pathways in cancer’ were included. There were 42 potential pathway proteins in the ‘pathways in cancer’ (Figure 5(a)) (the pathway proteins in all 38 pathways are listed in Sup Table 1). Meanwhile, Venn diagram analysis revealed that 399 genes shared miR-106a-5p targets in five databases (miRanda, TargetScan, PicTar, miRmap and microT) (Figure 5(b)). Venn diagram analysis showed that STAT3 was a frequent target of miR-106a-5p (Figure 5(c)), and STAT3 contained sites that paired with miR-106a-5p bases (Figure 5(d)). Thus, we carried out a dual luciferase activity experiment, and the results further verified that STAT3 was the target gene of miR-106a-5p (p < 0.001, Figure 5(e)). The STAT3 mRNA level in BC cells was detected by RT-PCR. The upregulation of miR-106a-5p significantly inhibited STAT3 mRNA levels (p < 0.001, Figure 5(f)). Importantly, several studies have found that STAT3 acts as an upstream regulator of HIF-1α and promotes its activation. Afterward, the expression levels of HIF-1α and STAT3 were measured by western blot. The results demonstrated that, compared with the control group, the HIF-1α and STAT3 levels in the VCAN-AS1 group were significantly upregulated, while the HIF-1α and STAT3 levels in the miR-106a-5p group were remarkably downregulated (p < 0.01, Figure 5(g,h)). Interestingly, the HIF-1α and STAT3 levels in the VCAN-AS1 + miR-106a-5p group were notably upregulated compared with those in the miR-106a-5p group (p < 0.01, Figure 5(g,h)). Hence, VCAN-AS1/miR-106a-5p modulates BC development by regulating the STAT3/HIF-1α pathway.

(a). Through mirPath v.3 (http://snf-515788.vm.okeanos.grnet.gr/index.php?r=mirpath), we predicted the potential signaling pathways of miR-106a-5p, including pathways in cancer (containing 42 genes). (b) Use starbase to predict the potential molecular targets of miR-106a-5p, and analyze the miR-106a-5p shared by five databases (miRanda, targetscan, pictar, miRmap and microT) using Wayne map target. (c). Analysis of the common target predicted by miR-106a-5p in mirPath v.3 and starbase using Wayne diagrams. (d) STAT3 contains base-pairing sites with miR-106a-5p. (e) The dual luciferase activity experiment was used to verify the targeting relationship between STAT3 and miR-106a-5p. (f) The mRNA expression levels of STAT3 were detected via RT-PCR. (g-h) The levels of HIF-1α and STAT3 were detected by western blot. NS means *p* > 0.05, ** *p* < 0.01, *** *p* < 0.001 vs. control group, ## *p* < 0.01, ### *p* < 0.001 vs. VCAN-AS1 group, &&& *p* < 0.001 vs. miR-106a-5p group.

### 3.6. Activating the STAT3 pathway reverses miR-106a-5p-mediated antitumor effects

**Figure 6.**
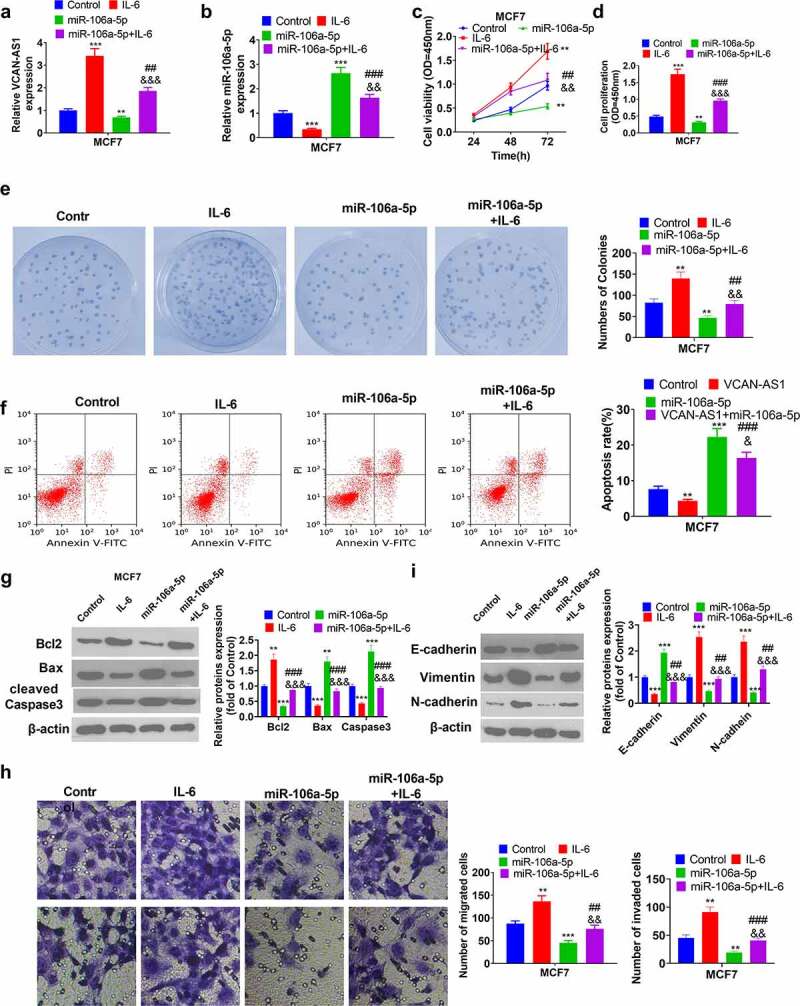
Activating the STAT3 pathway reverses miR-106a-5p-mediated antitumor effects

To further confirm that STAT3 is the downstream target of miR-106a-5p, we treated MCF7 cells with interleukin-6 (IL-6), the activator of the STAT3 pathway, according to previous studies [25,26]. Briefly, MCF7 cells were treated with IL-6 (100 ng/ml, No. SRP3096, Sigma) for 2 h. We observed that STAT3 was significantly activated by IL-6 (data not shown). The RT-PCR results showed that IL-6 enhanced VCAN-AS1 expression and reduced miR-106a-5p (compared with the control group, p < 0.001, Figure 6(a,b)). Then, we found that cell viability, proliferation, and colony formation increased in the IL-6 group, while that of the miR-106a-5p group decreased (compared with the control group) (p < 0.01, Figure 6(c,e)). Interestingly, compared with the miR-106a-5p group, cell viability and proliferation increased in the miR-106a-5p+IL-6 group (p < 0.05, Figure 6(c,e)). In addition, flow cytometry and western blot results showed that the IL-6 group and miR-106a-5p+IL-6 group had reduced cell apoptosis compared with the control group or miR-106a-5p group (p < 0.05, Figure 6(f,g)). Moreover, the migration, invasion and EMT of MCF7 cells were also aggravated by IL-6 treatment (compared with that in the control group or miR-106a-5p group, p < 0.01, Figure 6(h,i)). As a result, IL-6 reversed the inhibitory effects of miR-106a-5p on BC cells.

BC cells MCF7 were transfected with miR-106a-5p mimics and/or treated with IL-6. (a-b) RT-PCR was performed to measure the expression of VCAN-AS1 (a) and miR-106a-5p (b). (c) Cell viability was detected using the CCK8 method. (d) The cell proliferation ability was examined via the BrdU method. (e) The cell colony ability was examined via the colony formation method. (f) Flow cytometry detection of cell apoptosis rate. (g) The expression of apoptosis-related proteins Bcl-2, Caspase3 and Bax were measured by western blot. (h) Transwell test was employed to detect MCF7 cell migration and invasion ability. (i) Changes in EMT-related proteins (E-cadherin, Vimentin and N-cadherin) were measured via western blot. ** *p* < 0.01, *** *p* < 0.001 vs. control group, ## *p* < 0.01, ### *p* < 0.001 vs. IL-6 group, & *p* < 0.05, && *p* < 0.01, &&& *p* < 0.001 vs. miR-106a-5p group.

### 3.7. Overexpression of VCAN-AS1 promotes tumor growth and EMT by regulating the miR-106a-5p/STAT3/HIF-1α pathway

**Figure 7.**
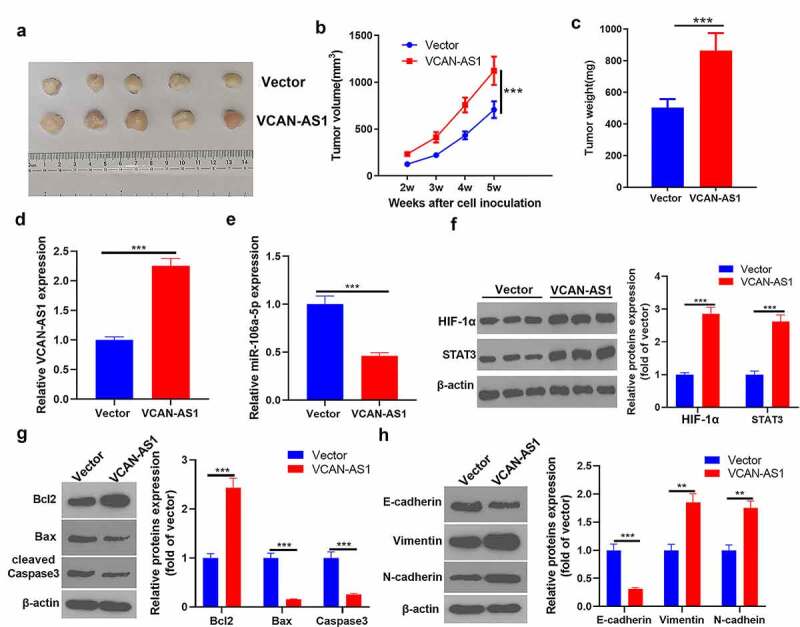
Overexpression of VCAN-AS1 promotes tumor growth and EMT by regulating the miR-106a-5p/STAT3/HIF-1α pathway

To clarify the regulatory effect of VCAN-AS1 on BC cell growth and metastasis, we conducted *in vivo* tumor experiments in nude mice. The results showed that the overexpression of VCAN-AS1 promoted the growth of BC cells (p < 0.001, Figure 7(a,c)). Molecular expression in tumor tissues was detected, and it was found that compared with the vector group, the levels of VCAN-AS1 and STAT3/HIF-1α in the VCAN-AS1 group were upregulated, while the levels of miR-106a-5p were downregulated (p < 0.001, Figure 7(d,f)). In addition, the expression of apoptosis- and EMT-related proteins was examined by western blot. We found that Bcl-2, vimentin, and N-cadherin expression was significantly upregulated in the VCAN-AS1 group, while the levels of Bax, caspase-3 and E-cadherin were considerably downregulated (p < 0.01, Figure 7(g,h)), indicating that VCAN-AS1 inhibited the apoptosis of cells *in vivo* and promoted EMT. Therefore, overexpression of VCAN-AS1 promotes tumor growth and EMT by regulating the miR-106a-5p/STAT3/HIF-1α pathway.

(a). MCF7 was taken to transfect VCAN-AS1 and vector respectively, and tumor xenograft model was constructed. (b) The tumor volume changes. (c) The mice were sacrificed at the fifth week, and the tumor mass was measured. (d-e) Detection of VCAN-AS1 (d) and miR-106a-5p (e) expression in tumor tissues by RT-PCR. (f-h) The expression of STAT3/HIF-1α (f), apoptosis-related protein (g) and EMT-related protein (h) was detected via western blot. ** means *p* < 0.01, *** means *p* < 0.001.

## 4. Discussion

In the present study, we investigated the expression characteristics of VCAN-AS1, a novel lncRNA, in BC tissues, as well as its role and mechanism in regulating BC development. Our results suggested that VCAN-AS1 is increased in BC and is related to worse patient survival. Additionally, VCAN-AS1 works as a ceRNA by sponging miR-106a-5p and activating the STAT3/HIF-1α pathway, thereby promoting the proliferation and metastasis of BC cells.

As the most common type of malignant tumor that harms women’s health, the high rate of metastasis in BC is a prominent reason. Data show that approximately 30%–40% of BC patients develop micrometastatic BC, and metastatic BC is often incurable [27]. The survival time of BC patients with distant metastasis is approximately 2–3 years, while only 5–10% of patients have a survival time of more than 5 years [27,28]. EMT is a process in which epithelial cells lose their polarity and reorganize the cytoskeleton into a migratory mesenchymal phenotype. By losing the epithelial phenotype, EMT tumor cells acquire the ability to move, invade, and resist apoptosis so that tumor cells can break away from the primary tumor site [29]. The transfer of BC is accompanied by significant EMT, and the transfer of BC can be attenuated by regulating EMT [29]. For example, Astragalus polysaccharide (APS), the main component of the traditional Chinese Astragalus membranaceus medicine, exerts antitumor effects on BC by attenuating the migration, invasion and EMT of BC cells through the Wnt/β-catenin signaling pathway [30]. Therefore, effective regulation of EMT in BC cells reduces the progression of BC.

In recent decades, an increasing number of studies have found that the aberrant expression of various lncRNAs in BC is closely related to its occurrence, proliferation, and metastasis. For example, lncRNA OLBC15 is overexpressed in BC and enhances the viability, migration and metastasis of BC cells both *in vivo* and *in vitro* [31]. Moreover, SNHG6 is upregulated in BC tissues and cell lines, and knocking down SNHG6 decreases BC cell proliferation, migration and invasion both *in vitro* and *in vivo* [32]. Here, we found that VCAN-AS1 promoted BC cell proliferation and viability, inhibited cell apoptosis, and accelerated BC cell migration, invasion and EMT. This phenomenon indicated that VCAN-AS1 could be used as a prognostic indicator of BC and exerts a carcinogenic effect on BC. In fact, it was previously reported that VCAN-AS1 is increased in gastric cancer tissues and is related to the poor prognosis of gastric cancer patients. Meanwhile, VCAN-AS1 also negatively regulates TP53 expression by competitively binding with eIF4A3 [9]. These findings confirm the oncogenic effect of VCAN-AS1 in the regulation of tumor progression.

As another type of noncoding RNA molecule, microRNA is involved in many biological processes through gene regulation, including the regulation of many tumors. In BC, miRNA also has a significant regulatory effect. For example, miR-185-5p induces a higher level of apoptosis in BC cells by targeting Bcl-2 [33]. Additionally, miR-615-3p is upregulated in BC and enhances EMT and metastasis of BC by targeting the PICK1/TGFBRI axis [34]. As an essential miRNA, miR-106a-5p has a variety of biological functions. For example, miR-106a-5p in combination with baicalein suppresses chondrocyte apoptosis in osteoarthritis [35]. miR-106a-5p targets FOXC1 to exert an inhibitory effect on endometriosis (EMS) development, likely through the PI3K/Akt/mTOR signaling pathway [36]. The function of miR-106a-5p is diverse in tumors. For example, miR-106a-5p plays a carcinogenic role in glioblastoma [37], colorectal cancer [38] and ovarian cancer [39] but has an anticancer effect in renal cell carcinoma [40] and nasopharyngeal carcinoma [41]. Notably, miR-106a-5p was found to be a survival predictor and to inhibit BC development in several studies [42,43]. Here, we proved that miR-106a-5p was downregulated in BC tissues, and a low level of miR-106a-5p was related to worse patient survival. More critically, overexpression of miR-106a-5p inhibited BC cell proliferation, migration, invasion, and EMT and accelerated cell apoptosis. These findings further confirmed the effect of miR-106a-5p on cancer inhibition in BC.

Many studies have found that lncRNAs, as endogenous competitive RNAs, affect the regulation of miRNA expression, while the latter regulates protein expression by binding to the 3ʹ-UTR end of mRNA transcribed by targeted genes in the miRNA-lncRNA-mRNA regulatory network [44]. Previous studies have found that the circular RNA RHOT1 competitively inhibits miR-106a-5p expression, thus upregulating the expression of STAT3 and accelerating the progression of BC [45]. In the present study, we found a negative correlation between the expression of VCAN-AS1 and miR-106a-5p in BC tissues. In addition, the upregulation of VCAN-AS1 inhibited miR-106a-5p, while the dual luciferase activity experiment and RIP experiment confirmed that VCAN-AS1 could adsorb miR-106a-5p. Functionally, the upregulation of VCAN-AS1 largely reverses the tumor-suppressive effect mediated by miR-106a-5p. Therefore, we speculated that during the BC process, VCAN-AS1 was upregulated, acting as a ceRNA to inhibit the expression and function of miR-106a-5p, a tumor suppressor molecule, thus promoting the progression of BC as a cancer-promoting factor.

STAT3 is a signal transduction protein involved in the response of a large number of cytokines and growth factors in cells and is responsible for the regulation of a series of important physiological processes, such as cell growth, proliferation, differentiation and apoptosis [46]. Activation of the JAK/STAT3 pathway also accelerates BC progression. For example, JAK/STAT3 enhances BC stem cell self-renewal and chemoresistance by regulating fatty acid β-oxidation [47]. Note that noncoding RNAs also regulate tumor progression by regulating STAT3. For instance, lncRNA NEAT1 negatively regulates miR-124, which serves as a target of STAT3. The NEAT1/miR-124/STAT3 axis forms a feedback loop to facilitate the proliferation and cell cycle progression of BC cells [48]. On the other hand, hypoxia-inducible factor 1 (HIF-1), another vital transcription factor, becomes activated under hypoxic stress and then promotes the transcription of genes that are involved in crucial aspects of cancer biology, including angiogenesis, cell survival, glucose metabolism and invasion [49]. Interestingly, STAT3 acts as an upstream regulator of HIF-1α and promotes its activation. For example, epidermal growth factor (EGF) increases the activation of STAT3 through its phosphorylation, thus inducing HIF-1α upregulation and promoting the proliferation and metastasis of colorectal cancer cells [50]. Furthermore, elevated phosphorylated STAT3 promotes trastuzumab resistance in human epidermal growth factor receptor 2 (HER2)-overexpressing BC cells by upregulating HIF-1α levels and downregulating PTEN [51]. These studies prove that the STAT3/HIF-1α pathway plays a crucial role in modulating BC development. In our study, our bioinformatics analysis results suggested that miR-106a-5p was an upstream target of STAT3. Further experiments indicated that miR-106a-5p could downregulate the STAT3/HIF-1α pathway, while activating the STAT3 pathway reversed the miR-106a-5p-mediated antitumor effect. Therefore, VCAN-AS1 stimulated the STAT3/HIF-1α pathway by sponging miR-106a-5p.

## 5. Conclusion

In summary, our study is the first to show that VCAN-AS1 promotes BC cell proliferation, migration, and invasion and inhibits cell apoptosis, suggesting that it may serve as a potential target for BC therapy. Related mechanistic studies have confirmed that VCAN-AS1 increases the STAT3/HIF-1α pathway by targeted inhibition of miR-106a-5p, thus exerting a carcinogenic effect. This study provides a new theoretical basis for BC treatment.

## Supplementary Material

Supplemental Material

## References

[1] Sung H, Ferlay J, Siegel RL, et al. Global cancer statistics 2020: GLOBOCAN estimates of incidence and mortality worldwide for 36 cancers in 185 countries. CA Cancer J Clin. 2021;71(3):209–249. 33538338 10.3322/caac.21660

[2] Yang B, Ren G, Song E, et al. Current status and factors influencing surgical options for breast cancer in China: a nationwide Cross-Sectional survey of 110 hospitals. Oncologist. 2020;25(10):e1473–e80. 32333626 10.1634/theoncologist.2020-0001 PMC7543333

[3] Fahad Ullah M. Breast cancer: current perspectives on the disease status. Adv Exp Med Biol. 2019;1152:51–64. 31456179 10.1007/978-3-030-20301-6_4

[4] Fatica A, Bozzoni I. Long non-coding RNAs: new players in cell differentiation and development. Nat Rev Genet. 2014;15(1):7–21. 24296535 10.1038/nrg3606

[5] Guttman M, Rinn JL. Modular regulatory principles of large non-coding RNAs. Nature. 2012;482(7385):339–346. 22337053 10.1038/nature10887 PMC4197003

[6] Wang H, Feng L, Zheng Y, et al. LINC00680 promotes the progression of non-small cell lung cancer and functions as a sponge of miR-410-3p to enhance HMGB1 expression. Onco Targets Ther. 2020;13:8183–8196. 32904350 10.2147/OTT.S259232 PMC7455755

[7] Xiong X, Feng Y, Li L, et al. Long non‑coding RNA SNHG1 promotes breast cancer progression by regulation of LMO4. Oncol Rep. 2020;43:1503–1515. 32323846 10.3892/or.2020.7530 PMC7107776

[8] Han L, Yan Y, Zhao L, et al. LncRNA HOTTIP facilitates the stemness of breast cancer via regulation of miR-148a-3p/WNT1 pathway. J Cell Mol Med. 2020;24(11):6242–6252. 32307830 10.1111/jcmm.15261 PMC7294123

[9] Feng L, Li J, Li F, et al. Long noncoding RNA VCAN-AS1 contributes to the progression of gastric cancer via regulating p53 expression. J Cell Physiol. 2020;235(5):4388–4398. 31637706 10.1002/jcp.29315

[10] Wang J, Ding Y, Wu Y, et al. Identification of the complex regulatory relationships related to gastric cancer from lncRNA-miRNA-mRNA network. J Cell Biochem. 2020;121(1):876–887. 31452262 10.1002/jcb.29332

[11] Kabekkodu SP, Shukla V, Varghese VK, et al.. Clustered miRNAs and their role in biological functions and diseases. Biol Rev Camb Philos Soc. 2018;93:1955–1986. 29797774 10.1111/brv.12428

[12] Xie F, Zhang L, Yao Q, et al. TUG1 promoted tumor progression by sponging miR-335-5p and regulating CXCR4-mediated infiltration of Pro-Tumor immunocytes in CTNNB1-Mutated hepatoblastoma. Onco Targets Ther. 2020;13:3105–3115. 32341656 10.2147/OTT.S234819 PMC7166065

[13] McGuire A, Brown JA, Kerin MJ. Metastatic breast cancer: the potential of miRNA for diagnosis and treatment monitoring. Cancer Metastasis Rev. 2015;34(1):145–155. 25721950 10.1007/s10555-015-9551-7 PMC4368851

[14] Ma J, Wang W, Azhati B, et al. miR-106a-5p Functions as a tumor suppressor by targeting VEGFA in renal cell carcinoma. Dis Markers. 2020;2020:8837941. 33224312 10.1155/2020/8837941 PMC7669356

[15] Guo J, Dou D, Zhang T, et al. HOTAIR promotes cisplatin resistance of osteosarcoma cells by regulating cell proliferation, invasion, and apoptosis via miR-106a-5p/STAT3 axis. Cell Transplant. 2020;29:963689720948447. 32757663 10.1177/0963689720948447 PMC7563817

[16] Wang M, Wang W, Ding J, et al. Downregulation of Rab17 promotes cell proliferation and invasion in non-small cell lung cancer through STAT3/HIF-1α/VEGF signaling. Thorac Cancer. 2020;11(2):379–388. 31841274 10.1111/1759-7714.13278 PMC6997001

[17] Banerjee K, Resat H. Constitutive activation of STAT3 in breast cancer cells: a review. Int J Cancer. 2016;138(11):2570–2578. 26559373 10.1002/ijc.29923 PMC4801660

[18] Hajizadeh F, Okoye I, Esmaily M, et al. Hypoxia inducible factors in the tumor microenvironment as therapeutic targets of cancer stem cells. Life Sci. 2019;237:116952. 31622608 10.1016/j.lfs.2019.116952

[19] Höckel M, Vaupel P. Biological consequences of tumor hypoxia. Semin Oncol. 2001;28:36–41. 11395851

[20] Schumacker PT. Hypoxia-Inducible Factor-1 (HIF-1). Crit Care Med. 2005;33(Suppl):423–425. 10.1097/01.ccm.0000191716.38566.e0 16340411

[21] Xu Q, Briggs J, Park S, et al. Targeting Stat3 blocks both HIF-1 and VEGF expression induced by multiple oncogenic growth signaling pathways. Oncogene. 2005;24(36):5552–5560. 16007214 10.1038/sj.onc.1208719

[22] Pawlus MR, Wang L, Hu CJ. STAT3 and HIF1α cooperatively activate HIF1 target genes in MDA-MB-231 and RCC4 cells. Oncogene. 2014;33(13):1670–1679. 23604114 10.1038/onc.2013.115 PMC3868635

[23] Dong L, Cao X, Luo Y, et al. A positive feedback loop of lncRNA DSCR8/miR-98-5p/STAT3/HIF-1α plays a role in the progression of ovarian cancer. Front Oncol. 2020;10:1713. 32984052 10.3389/fonc.2020.01713 PMC7492662

[24] Li G, Du P, He J, et al. CircRNA circBACH1 (hsa_circ_0061395) serves as a miR-656-3p sponge to facilitate hepatocellular carcinoma progression through increasing SERBP1 expression. Biochem Biophys Res Commun. 2021;556:1–8. 33831787 10.1016/j.bbrc.2021.03.136

[25] Cao C, Zhao G, Yu W, et al. Activation of STAT3 stimulates AHSP expression in K562 cells. Sci China Life Sci. 2014;57(5):488–494. 24740453 10.1007/s11427-014-4652-z

[26] Li SC, Lee CC, Hsu CM, et al. IL-6 induces haptoglobin expression through activating STAT3 in human head and neck cancer. J Oral Pathol Med. 2020;49(1):49–54. 31478236 10.1111/jop.12958

[27] Cardoso F, Fallowfield L, Costa A, et al. Locally recurrent or metastatic breast cancer: ESMO Clinical Practice Guidelines for diagnosis, treatment and follow-up. Ann Oncol. 2011;22(Suppl 6):vi25–30. 21908499 10.1093/annonc/mdr372

[28] Kolak A, Kamińska M, Sygit K, et al. Primary and secondary prevention of breast cancer. Ann Agric Environ Med. 2017;24(4):549–553. 29284222 10.26444/aaem/75943

[29] Bill R, Christofori G. The relevance of EMT in breast cancer metastasis: correlation or causality? FEBS Lett. 2015;589(14):1577–1587. 25979173 10.1016/j.febslet.2015.05.002

[30] Yang S, Sun S, Xu W, et al. Astragalus polysaccharide inhibits breast cancer cell migration and invasion by regulating epithelial‑mesenchymal transition via the Wnt/β‑catenin signaling pathway. Mol Med Rep. 2020;21:1819–1832. 32319619 10.3892/mmr.2020.10983 PMC7057808

[31] Deng C, Zhang B, Zhang Y, et al. A long non-coding RNA OLBC15 promotes triple-negative breast cancer progression via enhancing ZNF326 degradation. J Clin Lab Anal. 2020;34(8):e23304. 32329931 10.1002/jcla.23304 PMC7439339

[32] Lv P, Qiu X, Gu Y, et al. Long non-coding RNA SNHG6 enhances cell proliferation, migration and invasion by regulating miR-26a-5p/MAPK6 in breast cancer. Biomed Pharmacother. 2019;110:294–301. 30522015 10.1016/j.biopha.2018.11.016

[33] Değerli E, Torun V, Cansaran-Duman D. miR-185-5p response to usnic acid suppresses proliferation and regulating apoptosis in breast cancer cell by targeting Bcl2. Biol Res. 2020;53(1):19. 32366289 10.1186/s40659-020-00285-4 PMC7197166

[34] Lei B, Wang D, Zhang M, et al. miR-615-3p promotes the epithelial-mesenchymal transition and metastasis of breast cancer by targeting PICK1/TGFBRI axis. J Exp Clin Cancer Res. 2020;39(1):71. 32336285 10.1186/s13046-020-01571-5 PMC7183699

[35] Xiang Q, Wang J, Wang T, et al. Combination of baicalein and miR-106a-5p mimics significantly alleviates IL-1β-induced inflammatory injury in CHON-001 cells. Exp Ther Med. 2021;21(4):345. 33732318 10.3892/etm.2021.9776 PMC7903477

[36] Zhou X, Chen Z, Pei L, et al. MicroRNA miR-106a-5p targets forkhead box transcription factor FOXC1 to suppress the cell proliferation, migration, and invasion of ectopic endometrial stromal cells via the PI3K/Akt/mTOR signaling pathway. Bioengineered. 2021;12(1):2203–2213. 34082653 10.1080/21655979.2021.1933679 PMC8806537

[37] Li D, Wang Z, Chen Z, et al. MicroRNA-106a-5p facilitates human glioblastoma cell proliferation and invasion by targeting adenomatosis polyposis coli protein. Biochem Biophys Res Commun. 2016;481(3–4):245–250. 27815074 10.1016/j.bbrc.2016.10.132

[38] Liu J, Huang Y, Wang H, et al. MiR-106a-5p promotes 5-FU resistance and the metastasis of colorectal cancer by targeting TGFβR2. Int J Clin Exp Pathol. 2018;11:5622–5634. 31949649 PMC6963073

[39] Chao H, Zhang M, Hou H, et al. HOTAIRM1 suppresses cell proliferation and invasion in ovarian cancer through facilitating ARHGAP24 expression by sponging miR-106a-5p. Life Sci. 2020;243:117296. 31935390 10.1016/j.lfs.2020.117296

[40] Pan YJ, Wei LL, Wu XJ, et al. MiR-106a-5p inhibits the cell migration and invasion of renal cell carcinoma through targeting PAK5. Cell Death Dis. 2017;8(10):e3155. 29072688 10.1038/cddis.2017.561 PMC5680926

[41] Zheng YJ, Zhao JY, Liang TS, et al. Long noncoding RNA SMAD5-AS1 acts as a microRNA-106a-5p sponge to promote epithelial mesenchymal transition in nasopharyngeal carcinoma. Faseb J. 2019;33(11):12915–12928. 31557058 10.1096/fj.201900803R PMC6902713

[42] Xing L, Tang X, Wu K, et al. LncRNA HAND2-AS1 suppressed the growth of triple negative breast cancer via reducing secretion of MSCs derived exosomal miR-106a-5p. Aging (Albany NY). 2020;13(1):424–436. 33290256 10.18632/aging.202148 PMC7835037

[43] Li M, Zhou Y, Xia T, et al. Circulating microRNAs from the miR-106a-363 cluster on chromosome X as novel diagnostic biomarkers for breast cancer. Breast Cancer Res Treat. 2018;170(2):257–270. 29557526 10.1007/s10549-018-4757-3 PMC5999170

[44] Li JP, Xiang Y, Fan LJ, et al. Long noncoding RNA H19 competitively binds miR-93-5p to regulate STAT3 expression in breast cancer. J Cell Biochem. 2019;120(3):3137–3148. 30256448 10.1002/jcb.27578

[45] Zhang H, Ge Z, Wang Z, et al. Circular RNA RHOT1 promotes progression and inhibits ferroptosis via mir-106a-5p/STAT3 axis in breast cancer. Aging (Albany NY). 2021;13(6):8115–8126. 33686957 10.18632/aging.202608 PMC8034942

[46] Wu M, Song D, Li H, et al. Negative regulators of STAT3 signaling pathway in cancers. Cancer Manag Res. 2019;11:4957–4969. 31213912 10.2147/CMAR.S206175 PMC6549392

[47] Wang T, Fahrmann JF, Lee H, et al. JAK/STAT3-Regulated fatty acid β-Oxidation is critical for breast cancer stem cell self-Renewal and chemoresistance. Cell Metab. 2018;27(1):136–50.e5. 29249690 10.1016/j.cmet.2017.11.001 PMC5777338

[48] Pang Y, Wu J, Li X, et al. NEAT1/miR‑124/STAT3 feedback loop promotes breast cancer progression. Int J Oncol. 2019;55:745–754. 31322202 10.3892/ijo.2019.4841

[49] Semenza GL. Targeting HIF-1 for cancer therapy. Nat Rev Cancer. 2003;3(10):721–732. 13130303 10.1038/nrc1187

[50] Zhao FL, Qin CF. EGF promotes HIF-1α expression in colorectal cancer cells and tumor metastasis by regulating phosphorylation of STAT3. Eur Rev Med Pharmacol Sci. 2019;23:1055–1062. 30779072 10.26355/eurrev_201902_16993

[51] Aghazadeh S, Yazdanparast R. Activation of STAT3/HIF-1α/Hes-1 axis promotes trastuzumab resistance in HER2-overexpressing breast cancer cells via down-regulation of PTEN. Biochim Biophys Acta Gen Subj. 2017;1861(8):1970–1980. 28499822 10.1016/j.bbagen.2017.05.009

